# Association between rapid renal function deterioration and cancer mortality in the elderly: A retrospective cohort study

**DOI:** 10.1002/cam4.5735

**Published:** 2023-03-07

**Authors:** I‐Ching Kuo, Yi‐Chi Chu, Yen‐Hsu Chen, Ta‐Chien Chan

**Affiliations:** ^1^ Kaohsiung Municipal Ta‐Tung Hospital Kaohsiung Taiwan; ^2^ Research Center for Humanities and Social Sciences, Academia Sinica Taipei Taiwan; ^3^ Division of Infectious Disease, Department of Internal Medicine, Kaohsiung Medical University Hospital Kaohsiung Medical University Kaohsiung Taiwan; ^4^ School of Medicine, Graduate Institute of Medicine, Sepsis Research Center, Center of Dengue Fever Control and Research Kaohsiung Medical University Kaohsiung Taiwan; ^5^ Department of Biological Science and Technology, College of Biological Science and Technology National Yang Ming Chiao Tung University Hsinchu Taiwan; ^6^ School of Medicine, College of Medicine National Sun Yat‐sen University; ^7^ Institute of Public Health, School of Medicine National Yang Ming Chiao Tung University Taipei Taiwan

**Keywords:** cancer mortality, eGFR, elderly, rapid kidney function decline

## Abstract

**Background:**

Kidney function is associated with clinical outcomes in patients with cancer.

**Objectives:**

This study aimed to assess the association between kidney function decline and cancer‐related mortality among community‐dwelling elderly individuals.

**Design:**

This was a retrospective longitudinal cohort study.

**Participants:**

The 61,988 participants were from an elderly health examination database in Taipei City from 2005 to 2012.

**Measurements:**

Multivariable logistic regression was used to assess the association between baseline covariates and rapidly deteriorating estimated glomerular filtration rate (eGFR). In addition, Cox proportional hazards model and the Fine–Gray model were used to quantify the effects of covariates on total cancer mortality and six specific cancer mortalities.

**Results:**

During the follow‐up period, 1482 participants died of cancer. Their baseline average eGFR was 73.8 ± 19.9 mL/min/1.73 m^2^, and 18.3% had rapid renal function decline (≥5 mL/min/1.73 m^2^ per year). Rapid renal function decline was positively related to age, baseline eGFR, proteinuria, hypertension, waist circumferences, high log triglyceride levels, and diabetes mellitus (DM) history. In Cox proportional hazard models, participants with rapid eGFR decline had an increased risk of cancer mortality [hazard ratio (95% CI): 1.97 (1.73, 2.24); *p* < 0.001] compared to those without rapid eGFR decline. In the analysis of site‐specific cancer mortality risk, rapid eGFR decline was associated with six site‐specific cancer mortality, namely gastrointestinal tract, hepatobiliary, lung, prostate, urinary tract, and hematological malignancies.

**Conclusions:**

Elderly individuals with rapid kidney function decline had higher cancer mortality risks. Serial assessments of dynamic changes in eGFR might provide information relevant for cancer prognosis.

## Graphical abstract

This study aimed to assess the association between kidney function decline and cancer‐related mortality among community‐dwelling elderly individuals. In Cox proportional hazard models, participants with rapid eGFR decline had an increased risk of cancer mortality compared to those without rapid eGFR decline. In the analysis of site‐specific cancer mortality risk, rapid eGFR decline was associated with six site‐specific cancer mortality, namely gastrointestinal tract, hepatobiliary, lung, prostate, urinary tract, and hematological malignancies.

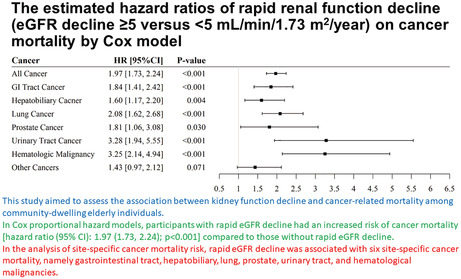

## 1 INTRODUCTION

Cancer incidence and mortality are steadily increasing worldwide, and are the leading cause of death.^1^, ^2^ In Taiwan, the four most common cancers are colorectal, lung, breast, and liver cancers. The number of deaths due to cancer was 50,161 which accounted for 28.98% of deaths due to all causes in 2020. The standardized cancer‐related mortality was 117.3 per 100,000 individuals in 2020.^3^ The majority, 85% of cancer occurs in patients aged more than 55 years. As the population of patients with cancer continues to age, the underlying comorbidities, such as hypertension, diabetes, cardiovascular disease, or coexistence of chronic disease, also have impact on the prognosis of a variety of cancers.^4^ Therefore, management of the underlying risks in addition to cancer itself is important to improve the clinical outcomes.

The prevalence of kidney dysfunction is high among cancer patients. This is partly because of age‐associated kidney dysfunction and chronic comorbidities, as cancer incidence increases with age. The Renal Insufficiency and Anticancer Medications (IRMA) study in France showed that 52.9% of 4684 patients with cancer had an estimated glomerular filtration rate (eGFR) of <90 mL/min/1.73 m^2^, which increased to 74.1% in 721 patients with cancer aged >75 years based on the Modification of Diet in Renal Disease (MDRD) formula.^5^ Additionally, several large prospective cohorts have found an association between a reduction in eGFR and higher cancer‐specific mortality.^6^, ^7^, ^8^, ^9^ Moreover, other studies have reported the association between proteinuria, which is an indicator of kidney dysfunction, and cancer‐related mortality.^9^, ^10^ The plausible underlying mechanism of this association may be a state of chronic inflammatory microenvironment^11^ and immunosuppression^12^ in chronic kidney disease (CKD), which promote cancer progression.

While CKD is a well‐established risk factor for all‐cause mortality, there has been growing evidence on the association between sequential changes in kidney function and mortality both above and below the eGFR of 60 mL/min per 1.73 m^2^.^13^, ^14^, ^15^, ^16^, ^17^ A few studies have also reported that rapidly declining eGFR increases all‐cause mortality rate.^13^, ^14^, ^15^ Longitudinal studies on kidney dysfunction and cancer‐related mortality in the elderly population are limited. Therefore, the present study aimed to assess the impact of kidney dysfunction on cancer‐related mortality among community‐dwelling elderly individuals.

## 2 METHODS

### 2.1 Data source

This community‐based cohort study used data from the Taipei City Elderly Health Examination Database. The study period covered from 2005 to 2012. The Taipei City Government provides free annual elderly health examinations in Taipei City. On average, around 42,000 elderly citizens aged ≥65 years each year can participate in the examinations, which accounts for 13% of the registered elderly population in Taipei City. All qualified participants can receive a health examination once annually. The examination included a standardized medical examination (clinical evaluation, oral examination, eye examination, anthropometric measurements, and biochemical tests) and questionnaires that addressed a variety of health‐related topics. This database is linked to Taiwan's National Death Registry from 2005 to 2012 by using the participants' identification number and personal information was encrypted by the data managers. Data were stored in the Taipei Geriatric Health Examination Database, and labels were de‐identified before release.

### 2.2 Selection of participants

**FIGURE 1:**
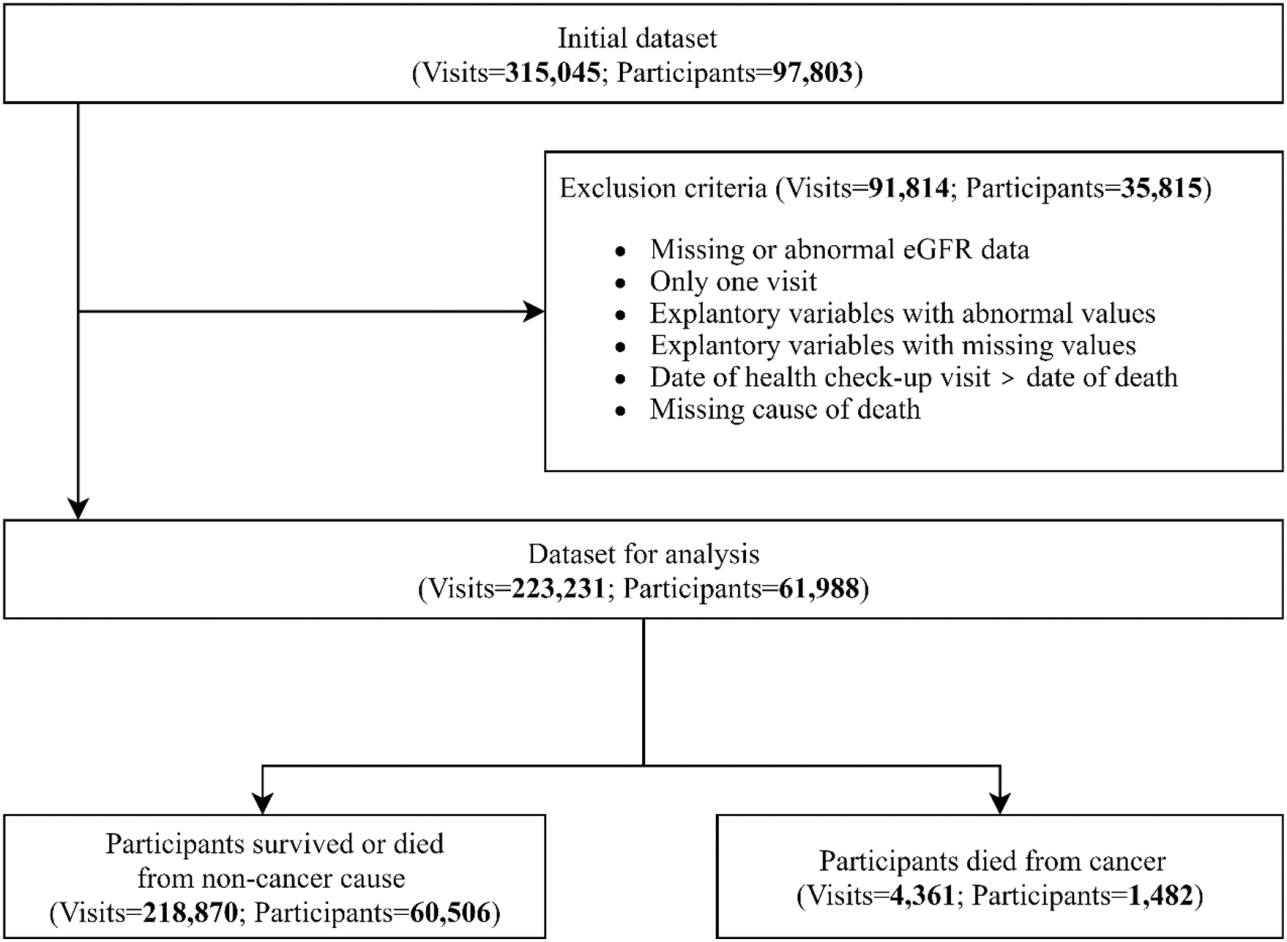
Flow chart of selecting participants.

This research was designed as a retrospective longitudinal cohort study based on the elderly health examination database in Taipei City. At the beginning, the database contained 315,045 health check‐up visits from 97,803 participants aged ≥65 years from 2005 to 2012. Each of the participants had different number of health check‐up visits. The selection process is shown in Figure 1. The renal function, assessed based on estimated glomerular filtration rate (eGFR), is one of our important explanatory variables. Thus, visits without serum creatinine data or with abnormal eGFR (≥200 mL/min/1.73 m^2^) were excluded (visits = 3957). The analysis required at least two time‐point measurements of the eGFR to compute the slope of the change. Therefore, we excluded 35,815 participants (91,814 visits) who had only one visit, explanatory variables with missing or abnormal values, and the date of health check‐up later than the date of death. Finally, we included 61,988 participants contributing to 223,231 visits. The endpoint outcome was defined as the deaths from malignant neoplasms registered in the death registry encoded by the ninth or tenth revision (the death year after 2009) of the international classification of diseases (ICD‐9 or ICD‐10).

### 2.3 Definition of covariates

The participant's baseline comorbidities, clinical conditions, and biochemical parameters were recorded. Trained case managers interviewed the participants using a structured questionnaire to obtain demographic information, lifestyle behaviors (such as cigarette smoking and alcohol drinking), pre‐existing comorbidities (such as diabetes and hypertension), and medications. Anthropometric parameters, including body height, weight, blood pressure, and waist circumference, were measured during the medical checkup. BMI was calculated as body weight (kg) divided by the square of the body height (m). The results of the biochemical tests of the blood samples were used for analysis. Hypertension was defined as systolic blood pressure (SBP) ≥140 mmHg, diastolic blood pressure (DBP) ≥90 mmHg, or the use of antihypertensive medication. Diabetes mellitus (DM) was defined as the use of medications. Biochemical data (white blood cells (WBCs), hemoglobin, albumin, triglyceride (TG), high‐density lipoprotein cholesterol (HDL‐C), creatinine, urine protein, and uric acid levels) were obtained from blood samples collected after midnight fasting. We examined the normality of biochemical values. Among all data distributions, only TG had a high skewness of 2.6. After the log transformation of TG (log TG), the skewness was reduced to 0.27, which is close to the normal distribution, with a skewness of 0.

We used the MDRD for the eGFR calculation formula. The formula is eGFR (mL/min/1.73 m^2^) = 186 × serum creatinine^−1.154^ × (age)^−0.203^ × (0.742 if female) × (1.212 if Black).^18^ The Kidney Disease: Improving Global Outcomes (KDIGO) 2012 clinical practice guideline for the evaluation and management of chronic kidney disease^19^ recommends the use of a GFR estimating equation to derive the estimated glomerular filtration rate (eGFR) from serum creatinine rather than relying on serum creatinine concentration alone. The KDIGO is used for classifying chronic kidney disease (CKD) into six stages based on the eGFR values: G1 (≥90 mL/min/1.73 m^2^), G2 (60–89 mL/min/1.73 m^2^), G3a (45–59 mL/min/1.73 m^2^), G3b (30–44 mL/min/1.73 m^2^), G4 (15–29 mL/min/1.73 m^2^), and G5 (<15 mL/min/1.73 m^2^). In this study, we classified eGFR (mL/min/1.73 m^2^) into four categories: ≥90, 60–89, 45–59, and <45. The number of participants with an eGFR of <45 was rare in the study population. Therefore, we did not classify them into a more detailed group with an eGFR of <45. The absolute annual rate of change in eGFR was calculated by fitting a least‐squares regression line to all measurements for each participant, where the slope of the regression line described the rate of change in eGFR. The rapid decline in kidney function was defined as an eGFR loss of ≥5 mL/min/1.73 m^2^ per year based on the KDIGO 2012 clinical practice guideline. A urine dipstick test for proteinuria was divided into negative, trace (+/−), 1+, and ≥2 +. We classified urine protein into three groups: “–“as a reference group, “+/−, +” as one group, and ++, +++, ++++ as a second group. Health risk behaviors, including cigarette smoking and alcohol drinking in the past half years, were treated as having or not.

The dependent variable was defined as death from any cancer or site‐specific cancer. Six specific types of cancer which had the highest number of deaths in this dataset were evaluated, namely lung, gastrointestinal, hepatobiliary, hematologic, prostate, and urinary tract cancers.

### 2.4 Statistical analyses

Baseline data of each participant was defined as the first non‐missing health‐checkup record during the whole study period. All participants were followed up from the date of the first health check‐up visit to the date of death, or the end of the study period (i.e., December 31, 2012), whichever came first. Study participants were censored because of death from causes other than the outcomes of interest, or the end of the study period. Since the dates of death were recorded in year‐month format, we imputed the day of death as the 15th of the month. For participants who died in the same month as the date of the last health check‐up visit, we imputed the day of death as the last day of the month.^20^

All analyses were performed with R software version 3.6.1,^21^ and we used the R package named “survival”^22^ for survival analysis. Descriptive statistics were used to summarize the participants' characteristics. Descriptive statistics were presented as mean ± standard deviation for continuous variables, and frequency (percentage) for categorical variables. Group differences were assessed using a one‐way ANOVA or *t*‐test for continuous variables and a chi‐squared test or Fisher's exact test for categorical variables as appropriate. Multivariable logistic regression was used to assess the association between baseline covariates and a deteriorated eGFR. The covariates were the same for overall cancer‐related mortality and mortality due to the six specific cancers. We checked for multicollinearity by using the variance inflation factors (VIFs). The VIFs of all the variables in the logistic regression were <5. Cox proportional hazard models were used to quantify the association between renal function deterioration and all‐cancer mortality and six specific cancer mortalities. The elderly population usually has multiple comorbidities and the competing risk of death is high. Thus, we additionally applied Fine and Gray's model^23^ to estimate the hazard ratios conservatively using the R package named “cmprsk”.^24^ The hazard ratios for eGFR decline in relation to the selected types of cancer are presented as forest plots.

### 2.5 Ethics

Participants were enrolled, wherein we obtained signed informed consent and submitted it to the Taipei City Government, to attend the health examinations and record the data for research purposes. The study protocol was approved by the Institutional Review Board (IRB) of Biomedical Science Research, Academia Sinica (IRB no. AS‐IRB‐BM 15043). In this study, we applied anonymous secondary data for analysis, and thus, the requirement for consent was waived by the IRB.

## 3 RESULTS

### 3.1 Participant characteristics

In this study, we report that 4512 elderly individuals had all‐cause deaths, including 1482 who died due to cancer and 3030 who died due to other causes. Table 1 summarizes the baseline characteristics of 61,988 participants stratified into four groups according to the baseline eGFR. This cohort had a mean age of 72.7 ± 6.3 years, and 51.1% of the study population was male. Overall, 7180 (11.6%) participants had DM and 38,316 (61.8%) participants had HTN. Mean age and percentage of sex, DM, and HTN were significantly different across four eGFR strata (*p* < 0.001). Mean length of follow‐up was 68.0 ± 23.6 months, with 4512 (7.3%) all‐cause deaths and 1482 cancer deaths (2.4%). The percentage of participants with proteinuria (>2+) was 1.9% in those with eGFR ≥90 versus 9.5% in those with eGFR<45.

**TABLE 1:** Baseline characteristics of all the elderly participants stratified by baseline eGFR.

Baseline characteristics Mean ± SD or *n* (%)	Overall (*n* = 61,988)	Baseline eGFR strata				*p* value
		eGFR ≥90, (*n* = 8299)	60 ≤ eGFR <90, (*n* = 39,157)	45 ≤ eGFR <60, (*n* = 11,087)	eGFR <45, (*n* = 3445)	
Follow‐up time (months)	68.0 ± 23.6	64.3 ± 23.6	67.9 ± 23.9	70.6 ± 22.6	70.5 ± 22.9	
Demography						
Age (year)	72.7 ± 6.3	71.4 ± 6.2	72.5 ± 6.2	73.4 ± 6.3	75.1 ± 6.8	<0.001
Gender (%)						<0.001
Male	31,664 (51.1)	3966 (47.8)	20,307 (51.9)	5581 (50.3)	1810 (52.5)	
Female	30,324 (48.9)	4333 (52.2)	18,850 (48.1)	5506 (49.7)	1635 (47.5)	
Disease history						
DM (%)	7180 (11.6)	875 (10.5)	4101 (10.5)	1526 (13.8)	678 (19.7)	<0.001
HTN (%)	38,316 (61.8)	4923 (59.3)	24,033 (61.4)	7073 (63.8)	2287 (66.4)	<0.001
Anthropometry						
BMI (kg/m^2^)	24.2 ± 3.4	23.9 ± 3.5	24.1 ± 3.4	24.5 ± 3.4	24.6 ± 3.5	<0.001
WC (cm)	84.4 ± 8.9	83.5 ± 9.1	84.3 ± 8.9	85.0 ± 8.8	85.5 ± 9.2	<0.001
Biochemical parameters						
WBC (10^3^/uL)	5.9 ± 2.1	5.8 ± 2.0	5.9 ± 2.0	6.1 ± 1.9	6.4 ± 2.9	<0.001
Hb (g/dL)	13.7 ± 1.6	13.6 ± 1.5	13.8 ± 1.5	13.6 ± 1.6	12.9 ± 1.9	<0.001
Alb (g/dL)	4.4 ± 0.7	4.3 ± 0.8	4.4 ± 0.7	4.4 ± 0.8	4.3 ± 0.4	<0.001
TG (mg/dL)	123.3 ± 72.3	115.0 ± 69.3	120.0 ± 69.3	133.9 ± 77.9	146.8 ± 84.6	<0.001
HDL‐C (mg/dL)	53.2 ± 14.1	55.5 ± 14.4	53.7 ± 14.0	51.2 ± 13.7	47.4 ± 12.7	<0.001
UA (mg/dL)	5.9 ± 2.0	5.2 ± 1.7	5.8 ± 1.9	6.5 ± 2.1	7.4 ± 1.9	<0.001
Baseline eGFR (mL/min/1.73 m^2^)	73.8 ± 19.9	108.2 ± 13.7	75.4 ± 8.8	54.2 ± 4.2	35.1 ± 9.3	
Proteinuria (%)						<0.001
Negative (−)	52,325 (84.4)	7195 (86.7)	33,549 (85.7)	9078 (81.9)	2503 (72.7)	
Trace (+/−), +	7921 (12.8)	950 (11.4)	4735 (12.1)	1622 (14.6)	614 (17.8)	
++, +++, ++++	1742 (2.8)	154 (1.9)	873 (2.2)	387 (3.5)	328 (9.5)	
eGFR decline (%)						<0.001
<5 (mL/min/1.73 m^2^/year)	50,649 (81.7)	4322 (52.1)	32,858 (83.9)	10,300 (92.9)	3169 (92.0)	
≧5 (mL/min/1.73 m^2^/year)	11,339 (18.3)	3977 (47.9)	6299 (16.1)	787 (7.1)	276 (8.0)	
Health behaviors						
Cigarette smoking (%)	4678 (7.5)	532 (6.4)	3012 (7.7)	866 (7.8)	268 (7.8)	<0.001
Alcohol drinking (%)	11,380 (18.4)	1419 (17.1)	7273 (18.6)	2038 (18.4)	650 (18.9)	0.014
Overall and site‐specific cancer deaths (%)^a^						
All cancer	1482 (2.4)	167 (2.0)	906 (2.3)	279 (2.5)	130 (3.8)	<0.001
Lung cancer	393 (0.6)	41 (0.5)	239 (0.6)	83 (0.7)	30 (0.9)	0.040
Gastrointestinal (GI) tract cancer	344 (0.6)	32 (0.4)	227 (0.6)	58 (0.5)	27 (0.8)	0.042
Hepatobiliary cancer	264 (0.4)	34 (0.4)	160 (0.4)	50 (0.5)	20 (0.6)	0.487
Hematologic malignancy	126 (0.2)	21 (0.3)	61 (0.2)	33 (0.3)	11 (0.3)	0.006
Prostate cancer	89 (0.1)	10 (0.1)	58 (0.2)	8 (0.01)	13 (0.4)	<0.001
Urinary tract cancer	84 (0.1)	3 (0.04)	52 (0.1)	16 (0.1)	13 (0.4)	<0.001
Other cancers	182 (0.3)	26 (0.3)	109 (0.3)	31 (0.3)	16 (0.5)	0.269
All‐cause deaths (%)	4512 (7.3)	511 (6.2)	2660 (6.8)	860 (7.8)	481 (14.0)	<0.001

*Note*: Data are expressed as the mean ± standard deviation or count (percentage).

Abbreviations: Alb, Albumin; BMI, body mass index; DM, diabetes mellitus; eGFR, estimated glomerular filtration rate; Hb, hemoglobin; HDL‐C, high density lipoprotein cholesterol; HTN, hypertension; TG, triglyceride; UA, uric acid; WBC, white blood cell; WC, waist circumference.

^a^ The percentage is computed from the number of site‐specific cancer‐related deaths dividing the number of participants in the strata.

To elucidate the association between kidney function and survival status, we stratified the groups according to death categories; survivor, cancer death, and non‐cancer death (Table 2). Among those cancer deaths, the average eGFR was 70.6 ± 20.4 mL/min/1.73 m^2^, and 382 (25.8%) participants had rapid kidney function deterioration (eGFR declines ≥5 mL/min per 1.73 m^2^ per year) compared to 17.5% with rapid eGFR decline in the survivor group (*p* < 0.001). Among non‐cancer deaths, the average eGFR was 68.9 ± 21.5 mL/min/1.73 m^2^, and 874 (28.8%) participants had rapid kidney function deterioration. The participants in the non‐cancer group were older and had a higher percentage of participants with a history of diabetes (*p* < 0.001) and hypertension (*p* = 0.033) and worse baseline eGFR than those in the cancer‐death group.

**TABLE 2:** Baseline characteristics of all the elderly participants stratified by the survival/death categories.

Baseline characteristics Mean ± SD or *n* (%)	Survived (*n* = 57,476)	Died from cancer (*n* = 1482)	Died from other causes(*n* = 3030)	*p* value
Follow‐up time (months)	69.0 ± 23.6	55.8 ± 20.3	54.9 ± 20.7	
Demography				
Baseline age (year)	72.2 ± 6.0	76.8 ± 6.3	79.5 ± 6.8	0.262
Gender (%)				<0.001
Male	28,553 (49.7)	1036 (69.9)	2075 (68.5)	
Female	28,923 (50.3)	446 (30.1)	955 (31.5)	
Disease history				
DM (%)	7180 (11.6)	187 (12.6)	413 (13.6)	<0.001
HTN (%)	35,459 (61.7)	916 (61.8)	1941 (64.1)	0.033
Anthropometry				
BMI (kg/m^2^)	24.2 ± 3.4	24.1 ± 3.5	23.8 ± 3.6	<0.001
WC (cm)	84.3 ± 8.9	85.7 ± 9.1	85.2 ± 9.5	<0.001
Biochemical parameters				
WBC (10^3^/uL)	5.9 ± 2.0	6.1 ± 2.0	6.1 ± 2.6	<0.001
Hb (g/dL)	13.7 ± 1.6	13.6 ± 1.7	13.5 ± 1.8	<0.001
Alb (g/dL)	4.4 ± 0.7	4.3 ± 0.7	4.3 ± 0.4	<0.001
TG (mg/dL)	123.5 ± 72.1	120.9 ± 73.2	121.4 ± 75	0.128
HDL‐C (mg/dL)	53.3 ± 14	50.6 ± 13.4	51.4 ± 14.3	<0.001
UA (mg/dL)	5.9 ± 2.0	6.1 ± 1.6	6.1 ± 1.9	<0.001
Baseline eGFR (mL/min/1.73 m^2^)	74.1 ± 19.7	70.6 ± 20.4	68.9 ± 21.5	<0.001
Proteinuria (%)				<0.001
Negative (−)	48,966 (85.2)	1180 (79.6)	2179 (71.9)	
Trace (+/−), +	7106 (12.4)	236 (15.9)	579 (19.1)	
++, +++, ++++	1404 (2.44)	66 (4.5)	272 (8.98)	
eGFR decline (%)				
<5 (mL/min/1.73 m^2^/year)	47,393 (82.5)	1100 (74.2)	2156 (81.2)	<0.001
≧5 (mL/min/1.73 m^2^/year)	10,083 (17.5)	382 (25.8)	874 (28.8)	
Health behaviors				
Cigarette smoking (%)	4242 (7.4)	153 (10.3)	283 (9.3)	<0.001
Alcohol drinking (%)	10,478 (18.2)	315 (21.3)	587 (19.4)	0.004

*Note*: Data are expressed as the mean ± standard deviation or count (percentage).

Abbreviations: Alb, Albumin; BMI, body mass index; DM, diabetes mellitus; eGFR, estimated glomerular filtration rate; Hb, hemoglobin; HDL‐C, high density lipoprotein cholesterol; HTN, hypertension; TG, triglyceride; UA, uric acid; WBC, white blood cell; WC, waist circumference.

### 3.2 Association between the baseline characteristics and rapid renal deterioration in all elderly participants

We found that rapid renal deterioration was associated with metabolic factors and baseline eGFR in the Multivariable logistic regression models (Table 3). Increasing age was associated with rapid eGFR decline in all four groups stratified by baseline eGFR. Overall, having HTN, increased waist circumference but not BMI, having high triglyceride level and the history of DM were all associated with higher odds of rapid eGFR decline. Besides, having proteinuria was associated with rapid renal function decline, particularly in those with lower baseline eGFR. On the contrary, albumin and hemoglobin were negatively associated with rapid renal function decline. The associations between gender and rapid renal function decline were not consistent in the higher and lower eGFR groups. In the group of eGFR ≥ 60 mL/min/1.73 m^2^, females had higher risk of rapid decline (eGFR ≥ 90, OR: 1.54, 95% CI: 1.38–1.71; 60 ≤ eGFR<90, OR: 1.27, 95% CI: 1.19–1.36). However, in the group of eGFR <60 mL/min/1.73 m^2^, males had higher risk of rapid decline (45 ≤ eGFR<60, OR: 0.68, 95% CI: 0.57, 0.81; eGFR<45, OR: 0.68, 95% CI: 0.50, 0.92).

**TABLE 3:** Odds ratios of rapid renal function decline (reduction in eGFR ≥5 vs. <5 mL/min/1.73 m^2^/year) in relation to baseline characteristics of elderly participants, overall and by baseline eGFR status.

Variables	Overall		Baseline eGFR strata							
			eGFR ≥90, (*n* = 8299)		60 ≤ eGFR <90, (*n* = 39,157)		45 ≤ eGFR <60, (*n* = 11,087)		eGFR <45, (*n* = 3445)	
	OR (95%CI)	*p*‐value	OR (95%CI)	*p*‐value	OR (95%CI)	*p*‐value	OR (95%CI)	*p*‐value	OR (95%CI)	*p*‐value
Age (years)	1.05 (1.04, 1.05)	<0.001	1.04 (1.04, 1.05)	<0.001	1.04 (1.03, 1.04)	<0.001	1.03 (1.02, 1.04)	<0.001	1.05 (1.03, 1.07)	<0.001
Female (ref.: male)	1.13 (1.07, 1.19)	<0.001	1.54 (1.38, 1.71)	<0.001	1.27 (1.19, 1.36)	<0.001	0.68 (0.57, 0.81)	<0.001	0.68 (0.50, 0.92)	0.012
HTN (yes/no)	1.08 (1.03, 1.13)	0.002	1.04 (0.95, 1.14)	0.429	1.08 (1.02, 1.14)	0.008	1.10 (0.94, 1.28)	0.249	0.87 (0.66, 1.15)	0.322
DM (yes/no)	1.08 (1.01, 1.16)	0.024	1.02 (0.88, 1.18)	0.791	1.07 (0.98, 1.17)	0.105	1.02 (0.82, 1.25)	0.869	1.29 (0.93, 1.76)	0.117
WC	1.01 (1.01, 1.01)	<0.001	1.01 (1.00, 1.01)	0.082	1.01 (1.01, 1.02)	<0.001	1.01 (1.00, 1.02)	0.106	1.03 (1.01, 1.05)	<0.001
BMI	1.00 (1.00, 1.01)	0.318	1.01 (1.00, 1.02)	0.171	1.00 (0.99, 1.00)	0.359	1.02 (0.99, 1.04)	0.166	1.02 (0.98, 1.06)	0.329
HDL‐C	1.00 (1.00, 1.00)	0.581	1.00 (1.00, 1.01)	0.018	1.00 (1.00, 1.00)	0.989	1.00 (0.99, 1.01)	0.861	1.01 (1.00, 1.02)	0.133
Log TG	1.08 (1.02, 1.13)	0.005	1.13 (1.03, 1.25)	0.014	0.98 (0.92, 1.05)	0.621	1.08 (0.91, 1.27)	0.394	1.11 (0.83, 1.48)	0.467
Alb	0.92 (0.86, 0.97)	0.009	0.90 (0.79, 1.00)	0.115	0.93 (0.85, 0.99)	0.054	0.84 (0.67, 1.02)	0.148	0.89 (0.65, 1.22)	0.467
Hb	0.94 (0.93, 0.96)	<0.001	0.94 (0.91, 0.98)	<0.001	0.97 (0.95, 0.99)	0.004	0.91 (0.86, 0.95)	<0.001	0.81 (0.75, 0.87)	<0.001
Baseline eGFR	1.05 (1.05, 1.05)	<0.001	—	—	—	—	—	—	—	—
Proteinuria	2.07 (1.95, 2.19)	<0.001	1.56 (1.37, 1.77)	<0.001	1.89 (1.77, 2.02)	<0.001	2.42 (2.06, 2.83)	<0.001	4.04 (3.10, 5.29)	<0.001
Cigarette smoking (yes/no)	1.02 (0.93, 1.11)	0.657	0.99 (0.82, 1.19)	0.911	1.02 (0.91, 1.13)	0.765	0.83 (0.62, 1.09)	0.192	1.34 (0.85, 2.05)	0.192
Alcohol drinking(yes/no)	0.99 (0.93, 1.05)	0.793	0.86 (0.76, 0.98)	0.019	1.02 (0.95, 1.10)	0.589	1.02 (0.84, 1.24)	0.813	1.09 (0.78, 1.50)	0.616

Abbreviations: Alb, Albumin; BMI, body mass index; CI, confidence interval; DM, diabetes mellitus; eGFR, estimated glomerular filtration rate; Hb, hemoglobin; HDL‐C, high density lipoprotein cholesterol; HTN, hypertension; OR, odds ratio; TG, triglyceride; WC, waist circumference.

### 3.3 Association between rapid renal deterioration and cancer mortality

Table 4 shows the results from the Cox model, which analyzed the covariates of all 1482 cancer‐related deaths. Among all participants, rapid renal deterioration was associated with higher risk of total cancer mortality, with a hazard ratio (HR) of 1.97 (95% CI: 1.73, 2.24), and proteinuria was also positively associated with cancer mortality (HR: 1.23, 95% CI: 1.08, 1.40). Furthermore, in each eGFR group, participants with rapid renal deterioration and proteinuria still had an increased risk of total cancer mortality.

**TABLE 4:** Hazard ratios of total cancer mortality in relation to baseline characteristics of elderly participants, overall and by baseline eGFR status.

Multivariable	Overall		Baseline eGFR strata							
			eGFR ≥90, (*n* = 8299)		60 ≤ eGFR <90, (*n* = 39,157)		45 ≤ eGFR <60, (*n* = 11,087)		eGFR <45, (*n* = 3445)	
Variables	HR (95%CI)	*p*‐value	HR (95%CI)	*p*‐value	HR (95%CI)	*p*‐value	HR (95%CI)	*p*‐value	HR (95%CI)	*p*‐value
Rapid renal function decline	1.97 (1.73, 2.24)	<0.001	1.56 (1.14, 2.15)	0.006	2.00 (1.72, 2.32)	<0.001	1.80 (1.17, 2.79)	0.008	1.86 (0.99, 3.48)	0.054
Age (years)	1.08 (1.07, 1.09)	<0.001	1.07 (1.05, 1.10)	<0.001	1.09 (1.07, 1.10)	<0.001	1.08 (1.06, 1.10)	<0.001	1.05 (1.03, 1.08)	<0.001
Female (ref.: male)	0.58 (0.51, 0.66)	<0.001	0.48 (0.32, 0.70)	<0.001	0.58 (0.49, 0.69)	<0.001	0.64 (0.48, 0.86)	0.003	0.61 (0.40, 0.92)	0.018
DM (yes/no)	1.05 (0.89, 1.22)	0.579	1.49 (0.96, 2.32)	0.075	1.18 (0.96, 1.44)	0.111	0.83 (0.57, 1.19)	0.302	0.57 (0.34, 0.96)	0.036
HTN (yes/no)	0.92 (0.83, 1.03)	0.138	1.04 (0.76, 1.42)	0.822	0.94 (0.82, 1.08)	0.390	0.78 (0.61, 0.99)	0.045	0.95 (0.66, 1.38)	0.799
WC	1.00 (0.99, 1.00)	0.559	1.00 (0.98, 1.01)	0.605	1.00 (0.99, 1.01)	0.490	1.00 (0.99, 1.02)	0.717	1.00 (0.98, 1.02)	0.847
BMI	1.00 (0.99, 1.02)	0.846	1.01 (0.96, 1.05)	0.780	1.00 (0.97, 1.02)	0.647	1.02 (0.98, 1.06)	0.325	1.00 (0.95, 1.06)	0.998
WBC	1.02 (1.00, 1.04)	0.015	1.03 (0.95, 1.11)	0.524	1.01 (0.99, 1.04)	0.270	1.04 (1.01, 1.08)	0.010	1.02 (0.97, 1.06)	0.477
Hb	0.93 (0.90, 0.97)	<0.001	0.98 (0.88, 1.09)	0.721	0.93 (0.89, 0.98)	0.004	0.97 (0.90, 1.05)	0.486	0.86 (0.79, 0.95)	0.003
HDL‐C	0.99 (0.99, 1.00)	0.009	1.00 (0.99, 1.01)	0.673	0.99 (0.99, 1.00)	0.014	1.00 (0.99, 1.01)	0.693	0.99 (0.97, 1.00)	0.086
Log TG	0.91 (0.81, 1.03)	0.133	0.65 (0.46, 0.93)	0.018	0.89 (0.77, 1.04)	0.142	1.12 (0.86, 1.46)	0.406	1.12 (0.77, 1.63)	0.565
Alb	0.91 (0.79, 1.06)	0.219	0.97 (0.66, 1.42)	0.864	0.97 (0.83, 1.12)	0.659	0.86 (0.60, 1.24)	0.427	0.75 (0.50, 1.13)	0.17
UA	1.00 (0.98, 1.03)	0.923	0.93 (0.82, 1.06)	0.288	1.02 (0.99, 1.04)	0.161	0.97 (0.90, 1.04)	0.328	1.00 (0.91, 1.09)	0.915
Baseline eGFR	0.99 (0.99, 1.00)	<0.001	—	—	—	—	—	—	—	—
Proteinuria	1.23 (1.08, 1.40)	0.001	1.20 (0.80, 1.79)	0.385	1.12 (0.95, 1.33)	0.188	1.44 (1.09, 1.91)	0.01	1.54 (1.05, 2.25)	0.027
Cigarette smoking (yes/no)	1.13 (0.95, 1.34)	0.173	0.58 (0.28, 1.18)	0.134	1.22 (0.99, 1.51)	0.058	0.91 (0.59, 1.39)	0.66	1.55 (0.91, 2.65)	0.109
Alcohol drinking (yes/no)	1.04 (0.91, 1.18)	0.565	1.19 (0.81, 1.74)	0.379	0.99 (0.84, 1.17)	0.878	1.15 (0.86, 1.54)	0.358	1.03 (0.67, 1.58)	0.893

Abbreviations: Alb, Albumin; BMI, body mass index; CI, confidence interval; DM, diabetes mellitus; eGFR, estimated glomerular filtration rate; Hb, hemoglobin; HDL‐C, high density lipoprotein cholesterol; HR, hazard ratio; HTN, hypertension; TG, triglyceride; UA, uric acid; WBC, white blood cell; WC, waist circumference.

**FIGURE 2:**
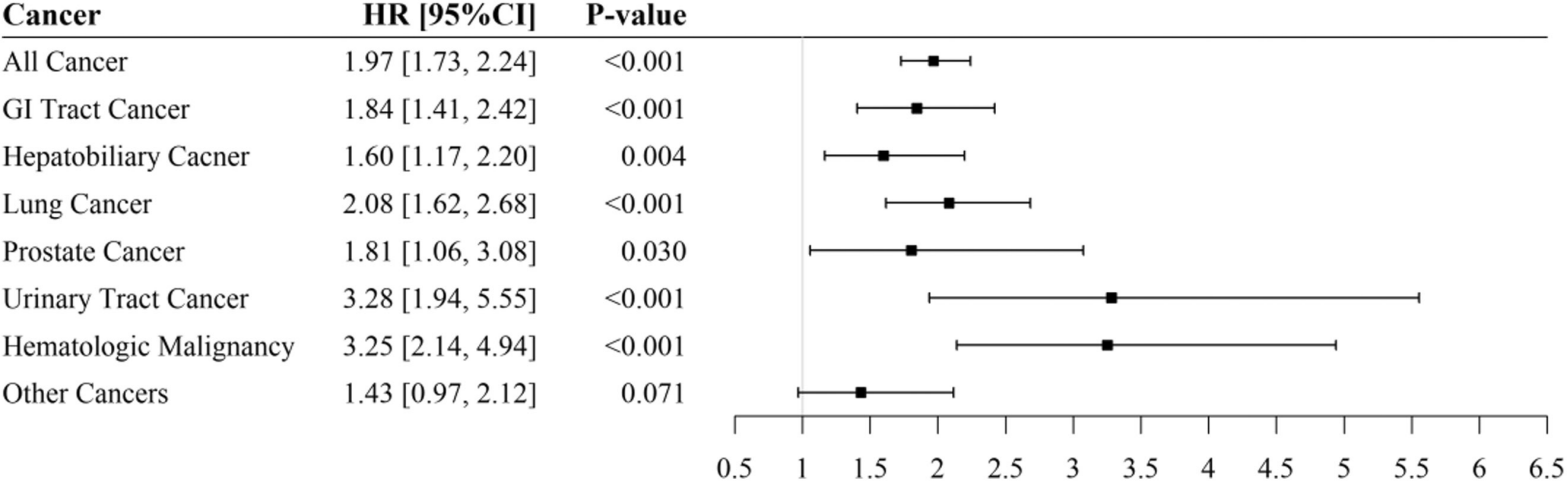
The estimated hazard ratios of rapid renal function decline (eGFR decline ≥5 vs. <5 mL/min/1.73 m^2^/year) on cancer mortality by Cox model. Adjusted for age, gender, DM, HTN, WC, BMI, WBC, Hb, HDL‐C, Log TG, Alb, UA, eGFR, proteinuria, cigarette smoking, alcohol drinking. Alb, Albumin; BMI, body mass index; CI, confidence interval; DM, diabetes mellitus; Hb, hemoglobin; HDL‐C, high density lipoprotein cholesterol; HR, hazard ratio; HTN, hypertension; TG, triglyceride; UA, uric acid; eGFR, estimated glomerular filtration rate; WBC, white blood cell; WC, waist circumference.

**FIGURE 3:**
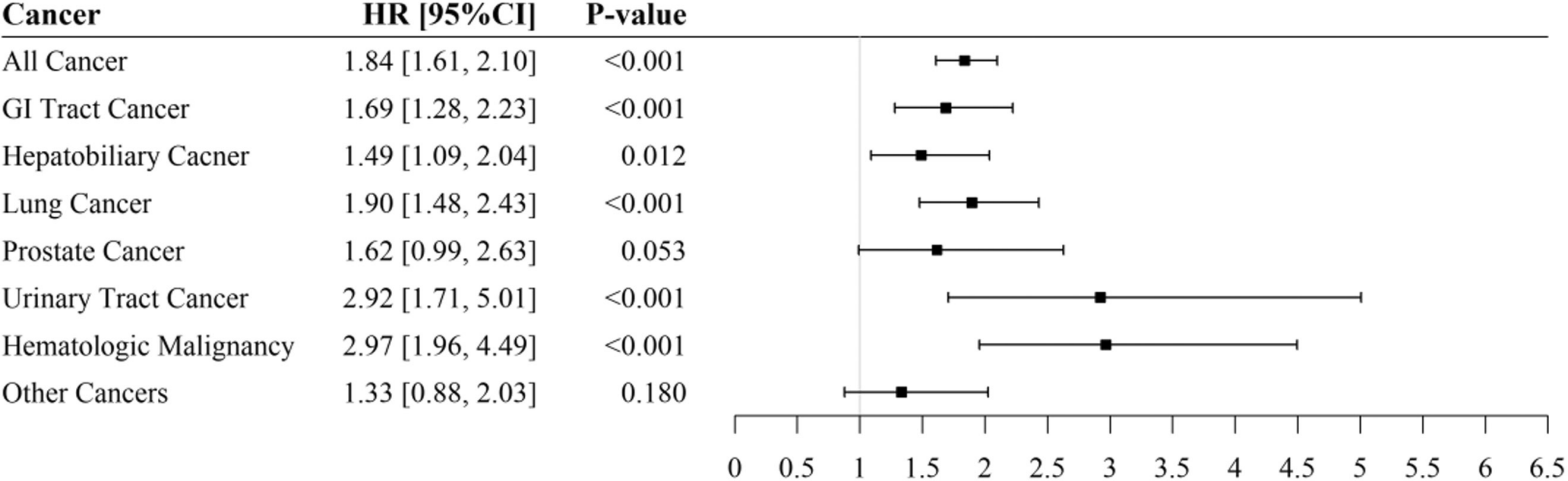
The estimated hazard ratios of rapid renal function decline (eGFR decline ≥5 vs. <5 mL/min/1.73 m^2^/year) on cancer mortality by Fine and Gray's model. Adjusted for age, gender, DM, HTN, WC, BMI, WBC, Hb, HDL‐C, Log TG, Alb, UA, eGFR, proteinuria, cigarette smoking, alcohol drinking. Alb, Albumin; BMI, body mass index; CI, confidence interval; DM, diabetes mellitus; eGFR, estimated glomerular filtration rate; Hb, hemoglobin; HDL‐C, high density lipoprotein cholesterol; HR, hazard ratio; HTN, hypertension; TG, triglyceride; UA, uric acid; WBC, white blood cell; WC, waist circumference.

In addition to total cancer mortality, Figure 2 shows the site‐specific risk of cancer death using the Cox model, and Figure 3 presents the conservative estimation from the Fine and Gray's model. As shown in Figure 2, rapid renal function decline was associated with an increased cancer mortality risk for gastrointestinal (HR: 1.84, 95% CI: 1.41, 2.42), hepatobiliary (HR: 1.60, 95% CI: 1.17, 2.20), lung (HR: 2.08, 95% CI: 1.62, 2.68), prostate (HR: 1.81, 95% CI: 1.06, 3.08), urinary tract (HR: 3.28, 95% CI: 1.94, 5.55), and hematological malignancies (HR: 3.25, 95% CI: 2.14, 4.94). In Figure 3, the Fine and Gray model also shows similar results, except for prostate cancer becoming marginally statistically significant (HR: 1.62, 95% CI: 0.99, 2.63).

## 4 DISCUSSION

In this longitudinal cohort of the elderly, rapidly declining renal function was associated with an increased risk of cancer mortality independent of metabolic components, baseline eGFR, and proteinuria. This finding remained consistent across subgroups of site‐specific cancers. To the best of our knowledge, this is the first large study that demonstrated the risk of rapidly declining renal function on cancer mortality in an elderly population.

Factors associated with rapid GFR decline in populations with normal renal function have been clinically studied. Disease processes underlying the rate of rapid renal decline and initially involved compartments of kidney (tubules, interstitium, vasculature, or glomeruli) are unknown. In a large cohort of 37,796 primary care patients with preserved kidney function, Koraishy et al. indicated that baseline eGFR, older age, African‐American race, unmarried status, lower neighborhood socioeconomic status, hypertension, type 2 diabetes, and smoking were associated with rapid eGFR decline (>5 mL/min/1.73 m^2^ per year).^25^ However, another longitudinal study comprising 72,521 healthy subjects aged ≥18 years old with mean eGFR 83.7 ± 14.7 mL/min/1.73 m^2^ showed that eGFR decline mainly depended on baseline eGFR, with faster decline with higher baseline eGFR values.^26^ According to a large cohort of 5168 participants in Taiwan, a prediction system was constructed to estimate CKD risk including clinical variables, such as age, body mass index, diastolic blood pressure, and proteinuria, as well as history of DM and stroke.^27^ Besides, in the general population with baseline eGFR of 80.7 mL/min/1.73 m^2^, the PREVEND study even found gender was a modifiable variable of predictors for renal function decline.^28^ More recently, other independent risk factors of accelerated renal function loss were reported including elevated serum uric acid level,^29^ metabolic syndrome,^30^ and high‐protein diet.^31^ Our cohort data presented a consistent finding in those aged ≥65 years old. In our analysis, old age, the presence of hypertension, increased waist circumference, having the history of DM, hypoalbuminemia, higher baseline eGFR, and proteinuria were related to rapid eGFR decline.

In the past decade, several studies have shown that loss of kidney function even with relatively well‐preserved kidney function at baseline was associated with adverse outcomes, including all‐cause mortality and poor renal outcomes. In 4380 elderly participants, Rifkin et al. found that rapid decline in eGFR (rate >3 mL/min/1.73 m^2^ per year) increased risk of mortality even though the high average eGFR was 69 mL/min/1.73 m^2^ at the end.^15^ The prospective analysis of data from the Atherosclerosis Risk in Communities (ARIC) Study reported that patients in the quartile with a steeper than average annual decline in eGFR had a higher risk for mortality.^14^ This was significantly important among patients with mildly and moderately reduced eGFR (30–89 mL/min per 1.73 m^2^). Recently, in a Chinese cohort of 37,691 participants aged ≥45 years with preserved kidney function, Guo et al. found that a greater decline in the eGFR over time was associated with the risk of mortality, independent of the initial eGFR.^32^ Since age is an important factor for the development of cancer, we focused on the risk factors of cancer mortality in an elderly population, especially the effect of kidney function. Furthermore, our observations regarding declining eGFR and elevated cancer mortality expanded to a larger community‐dwelling elderly population, which is different from previously published studies as mentioned before. Rapid decline of eGFR (≥5 mL/min/1.73 m^2^ per year) is associated with cancer mortality independently of other clinically relevant risk factors.

The causative mechanisms by which eGFR decline contributes to cancer mortality are not fully understood and are likely to be multifactorial. Decreasing renal function may be implicated in chronic inflammation, oxidative stress, and endothelial dysfunction. Inflammation and an increase in oxidative stress in turn may exploit underlying mutagenesis and genomic variability in cancer cells to stimulate cancer progression.^33^ Alternatively, when renal function progresses to advanced CKD, it is hypothesized that uremic toxins accumulation and microbial dysbiosis may lead to cancer development and progression.^34^, ^35^

Cancer is more prevalent in the elderly, as 60% of newly diagnosed cancers are in those aged 65 or older, as are 70% of all cancer deaths.^36^ Comorbid conditions possess many common risk factors predisposing one to cancer, such as obesity, smoking, lifestyle, chronic infection, or dysregulation of the immune system.^37^ Besides, comorbidities have impacts on cancer detection, treatment, and adherence, and tend to increase all‐cause mortality.^38^ Regarding kidney dysfunction in cancer, an important comorbidity, those who have rapid eGFR decline may suffer high levels of toxicity or complications from treatment, which reduces cancer‐specific survival. Moreover, rapid eGFR decline in cancer can be caused by chemotherapeutic drugs, malignant ureteral obstruction, nephrectomy, or paraneoplastic glomerulonephritis, all of which may lead to acute kidney injury, proteinuria, acid–base, or electrolytes imbalances.^39^ Our data also support the notion that rapid eGFR decline affects overall cancer mortality in the elderly who are more susceptible to nephrotoxic agents. It indicates that serial eGFR assessment is important for obtaining prognosis information.

Our study has several limitations. First, we were unable to obtain information on the time of definite diagnosis of cancer, which is a major limitation of this study. Disentangling the temporal order between cancer diagnosis and decreased eGFR was challenging. Therefore, we could not determine whether the treatment course changed the degree of kidney dysfunction, particularly in chemotherapy‐induced nephrotoxicity. Second, the estimated equation for kidney function may overestimate the eGFR because the elderly individuals with cancer often have cachexia and decreased muscle mass, which affects their creatinine concentration compared to healthy individuals. Third, although we adjusted the mortality‐related clinical risk factors, there may still be important residual confounders, such as the use of chemotherapeutic agents and cancer stages. Fourth, the cancer diagnosis was based on the ICD diagnosis as the major cause of death. Therefore, we may have underestimated the number of participants with cancer with missed cancer diagnosis in their death registry. Finally, due to unavailability of date of cancer diagnosis and uncertainty in serum creatinine in cancer patients, as well as irregular health check intervals of participants, we were unable to apply the longitudinal logistic models with the patient as the cluster or time‐dependent Cox models to explain time‐varying variables in mortality outcomes.

This study demonstrated that a rapid eGFR decline among the elderly individuals in communities is an independent risk factor for cancer mortality. In addition to baseline kidney function, serial assessments of dynamic changes in the eGFR may provide additional information to predict the prognosis. Future studies should examine the mechanisms through which kidney dysfunction affects tumor progression and relevant prevention strategies.

## AUTHOR CONTRIBUTIONS

**I‐Ching Kuo:** Conceptualization (lead); methodology (equal); writing – original draft (lead). **Yi‐Chi Chu:** Formal analysis (lead); visualization (lead). **Yen‐Hsu Chen:** Conceptualization (equal); writing – review and editing (equal). **Ta‐Chien Chan:** Conceptualization (equal); data curation (lead); funding acquisition (lead); methodology (lead); supervision (lead); writing – review and editing (lead).

## FUNDING INFORMATION

This research was supported by a grant from the Ministry of Science and Technology, Taiwan (MOST 111‐2121‐M‐001‐002). The funder had no role in study design, data collection and analysis, decision to publish, or preparation of the manuscript.

## CONFLICT OF INTEREST STATEMENT

The authors declare that they have no conflict of interest.

## ETHICS APPROVAL AND CONSENT TO PARTICIPATE

When participants were enrolled, Department of Health, Taipei City Government obtained signed informed consent from the participants. The study protocol was approved by the Institutional Review Board (IRB) of Biomedical Science Research, Academia Sinica (IRB no. AS‐IRB‐BM 15043). In this study, we applied anonymous secondary data for analysis, and thus, the requirement for consent was waived by the Institutional Review Board (IRB) of Biomedical Science Research, Academia Sinica. This study was performed in accordance with the Declaration of Helsinki and followed by the approved protocol.

## CONSENT FOR PUBLICATION

Not applicable.

## Data Availability

The data that support the findings of this study are available from the Department of Health, Taipei City Government but restrictions apply to the availability of these data, which were used under license for the current study, and so are not publicly available. Data are however available from the authors upon reasonable request and with permission of Health Promotion Division of the Department of Health, Taipei City Government.
